# Mitigating atherosclerosis: Integrating vaccines with gene targets

**DOI:** 10.1016/j.ahjo.2025.100588

**Published:** 2025-08-06

**Authors:** Alireza Haraj, Masoomeh Bakhshandeh, Nafiseh Shokri, Prand Shariat Rad, Ali Alyan, Zahra Chegini, Mohammad Ali Nazari, Seyed Parsa Seyedi Taji, Mohammad Najafi

**Affiliations:** a Clinical Biochemistry Department, Faculty of Medical Sciences, Iran University of Medical Sciences, Tehran, Iran; b Clinical Biochemistry Department, Faculty of Medical Sciences, Shahid Beheshti University of Medical Sciences, Tehran, Iran; c Student Research Committee, Kermanshah University of Medical Sciences, Tehran, Iran; d Microbial Biotechnology Research Center, Iran University of Medical Sciences, Tehran, Iran; e Student Research Committee, Faculty of Medical Sciences, Iran University of Medical Sciences, Tehran, Iran

**Keywords:** Atherosclerosis, Vaccine, Gene, Inflammation

## Abstract

The formation and progression of atherosclerotic plaques occur through cellular dysfunction and remodeling of the extracellular matrix in the sub-endothelial space of vessels. The immunity against specific antigens is suggested to mitigate the atherosclerosis process. Primarily, studies have suggested that certain antigens, such as ox-LDL, ApoB-100, CETP, PCSK9, HSP60, MHC-II-derived peptides, and interleukins, are involved in atherosclerosis. However, recognizing the intricate interplay between immune responses and the formation of arterial plaques is essential to optimize immunization against atherosclerosis. In this review, the roles of some genes were presented in triggering atherosclerotic plaque events. Furthermore, some immunization approaches are presented to target these genes. The studies suggested that vaccination against the progression of atherosclerosis is an essential and effective approach to reducing the high death rate in autoimmune diseases.

## Highlights

• Plaques of atherosclerosis occur through cellular dysfunction.
• Circulatory antigens and molecules are suggested for the production of vaccines against the atherosclerosis process.
• Vaccination is suggested as an effective approach to reducing the death rate in autoimmune diseases.

Atherosclerosis progresses through immune-cell dysfunction, extracellular matrix remodeling, and plaque growth in the sub-endothelial space of vessels. The immune cells are proposed as signal-generating focal points that stimulate and polarize other cells, including the vascular smooth muscle and endothelial cells in the plaque microenvironment. Dysfunction of these immune cells contributes to plaque instability, following vessel wall erosion and rupture. Suppressing immune-mediated inflammation is therefore a promising approach to control atherosclerosis. Notably, vaccination against specific atherosclerosis-related antigens is emerging as a potential preventive strategy. In this review, we discuss how immune responses drive plaque development and evaluate candidate antigens and immunization approaches to mitigate atherosclerosis.

## 1 Immune events including cellular dysfunctions and inflammatory mediators trigger the atherosclerosis process

### 1.1 Cellular dysfunctions

**Fig. 1:**
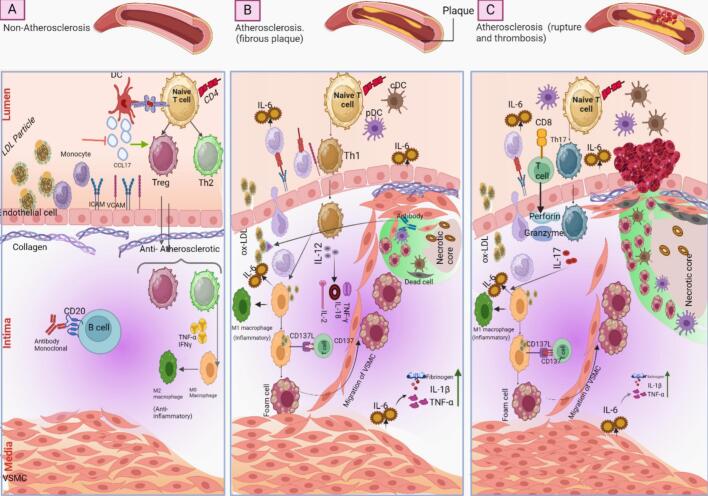
Inflammatory events in the vessel. **A**, Non-atherosclerotic vessel, trafficking of cells and other circulatory compounds through the endothelial layer. **B**, Atherosclerotic plaque. A primary plaque core forms in the sub-endothelial space. Then, it develops due to inflammation, cellular proliferation, and migration in the plaque microenvironment. **C**, Atherosclerosis plaque rupture. The cellular dysfunction and extracellular matrix remodeling progress the plaque rupture. https://www.biorender.com

It is well known that plaque formation, progression, and remodeling are associated with cellular responses in the atherosclerosis process (Fig. 1). Immune cells, including T cells, B cells, dendritic cells, monocytes, and macrophages, are vastly involved in the progression of atherosclerosis. Qiao et al. showed an association between immune reactions and lipid metabolism in mouse macrophages [1]. The dysfunctions of other cells are also reported in the plaque microenvironment [2]. Some studies have reported the roles of T cells in the development of inflammatory reactions. The CD4^+^ helper/inducer cells were reported to be more frequently present in the fibrous cap of plaque [3]. It is also shown that the CD4^+^ T cells respond to the lipid compounds and contribute to atherosclerosis [[4], [5], [6], [7]]. The vessel restenosis has also been reported to be involved with Th1 and Th2 cells [8], cytokines [9,10], and Regulatory T cells (Tregs) [11]. Furthermore, Th17 cells are correlated with the advanced stages of carotid artery stenosis [12]. The CD8^+^ T cells were also reported in the progression of atherosclerosis [13]. These cells contributed to the monocyte proliferation in the low-density lipoprotein receptor-deficient (LDLR^−/−^) mice [14]. It was also reported that CD137 is found in human atherosclerotic lesions and can accelerate the inflammatory events in the CD8^+^ cells of ApoE^−/−^ mice [15]. Furthermore, the CD8^+^ T cells trigger the inflammation in the plaque area, suggesting the advancement of atherosclerosis in CBL-B-deficient mice [16]. The secreted perforin and granzyme B by CD8^+^ T cells might contribute to the progression of unstable atherosclerotic plaques [17]. The B cells also play an important role in the progression of atherosclerosis [18]. Atherosclerotic lesions might contain antibodies that target specific epitopes [[19], [20], [21]]. Targeting the mature B cells with a specific monoclonal antibody against CD20 led to a notable decrease in the atherosclerosis process [22]. However, some studies reported that the B cells have a protective role in the progression of atherosclerosis. The transfer of B cells from older atherosclerotic ApoE−/− mice had a beneficial effect on the progression of atherosclerosis in mice that had undergone splenectomy [23]. Furthermore, the deficiency of B cells progressed the atherosclerosis process in LDLR −/− mice [24]. Moreover, activation of the B1a cells reduced the ox-LDL-related lesions and necrotic core size [25,26]. Other studies have also demonstrated the atheroprotective roles of the B cells [[27], [28], [29]]. The dendritic cells (DCs) were found to promote atherosclerosis [30]. Both the plasmacytoid (p) and conventional (C) DCs have been identified in the arteries and atherosclerotic plaques [30,31]. Blocking the CCL17 in DCs expanded Tregs and reduced the progression of atherosclerosis [32]. The removal of DC DNGR1 receptor decreased the presence of inflammatory cells in arterial plaques and restricted the progression of atherosclerosis in LDLR^−/−^ mice [33]. Disrupting the autophagy in DCs of LDLR^−/−^ mice increased the number of cells in the aorta, decreased the buildup of type 1 T helper cells, and mitigated the progression of the atherosclerosis process [34]. The antimicrobial peptides in atherosclerosis plaques also activated autoimmune responses in the pDCs [35]. However, using 120G8 mAb to deplete pDCs increased the accumulation of T cells in plaques and developed the progression of atherosclerosis lesions in LDLR^−/−^ mice [36]. The pDCs also enhanced the cytolytic effects of T cells [37]. The effects of hypercholesterolemia on the function of monocytes have also been approved in animal models [38]. The inhibition of leukocyte diapedesis decreased the development of atherosclerosis lesions [39]. Moreover, monocytes are polarized into macrophages and play a key role in the advancement of atherosclerosis [40]. The remodeling process relates to the function of macrophages in the plaque by controlling the synthesis of collagen and generating enzymes such as matrix metalloproteinase (MMPs) that can affect the stability of plaques [[41], [42], [43]]. Furthermore, the macrophage polarization affects the plaque phenotype [44], so the atherosclerosis process is improved by M1 macrophages [45].

### 1.2 Inflammatory mediators

Cytokines and growth factors are found in the atherosclerotic plaques [46]. TNF-α is reported in the edges of the inflammatory lesions [47], so that atherosclerosis diminished in the apoE-deficient mice with the disrupted TNF-α. The atherogenic role of TNF-α is related to the increase of some adhesion molecules such as vascular cell adhesion molecule-1 (VCAM-1) and intercellular adhesion molecule-1 (ICAM-1) [48]. However, some studies reported that the TNF p55 receptors protect mice against the development of atherosclerosis [49]. The inhibition of IL-1β using Canakinumab also decreased the cardiovascular events in patients [50]. This report also suggested that TNF-α can induce the synthesis of IL-1β [50]. Conversely, exposure to IL-1β leads to the development of coronary intima lesions in pigs [51]. IL-6 relates highly to the coronary artery calcium score. IL-6 elevates fibrinogen and other inflammatory cytokines such as IL-1β and TNF-α. It also enlarges the lesion size in the apoE-deficient mice [52]. On the other hand, the apoE-deficient mice with a lack of IL-6 exhibited the development of atherosclerotic plaques [53]. Thus, it is proposed that IL-6 levels are linked to a risk of heart attack [54]. These results also showed that normal IL-6 levels are necessary to regulate lipid balance [53]. IL-17 also promoted atherosclerosis through the increase of IL-6 in macrophages [55]. Moreover, the suppression of IL-17 in the apoE-deficient mice affected the IL-4 and IFN-γ values, resulting in the reduction of the atherosclerosis process [56]. In apoE /IL-17 A deficient mice, the vulnerable plaques are characterized by a decrease in the VSMCs and type I collagen levels [57]. Moreover, the mice with the suppressor of cytokine signaling 3 (SOCS3) deficiency demonstrated higher levels of IL-17 and induced the anti-inflammatory characteristics of macrophages [58]. The IL-12 also promoted the Th1 immune responses by producing cytokines such as IL-2, IFN-γ, and IL-18 [55]. Moreover, the changes in the IL-12 family are strongly related to the formation of carotid atherosclerotic plaque [59]. It was also reported that inhibition of IL-12 reduces the development of atherosclerosis in the LDLR^−/−^ mice [60]. Exposing the CD4^+^/CD28^+^ T cells to IL-12 showed an increased number of T cells [61]. Furthermore, the antagonist of the neuropeptide Y1 receptor increases the progression of atherosclerosis by the production of IL-12 in apoE-deficient mice [62]. Daily administration of IL-12 to apoE-deficient mice resulted in the development of atherosclerosis [63]. These findings suggested that targeting the inflammatory mediators produced by immune cells may help suppress atherosclerosis progression.

## 2 Persistent inflammatory events are observed in autoimmune diseases

**Fig. 2:**
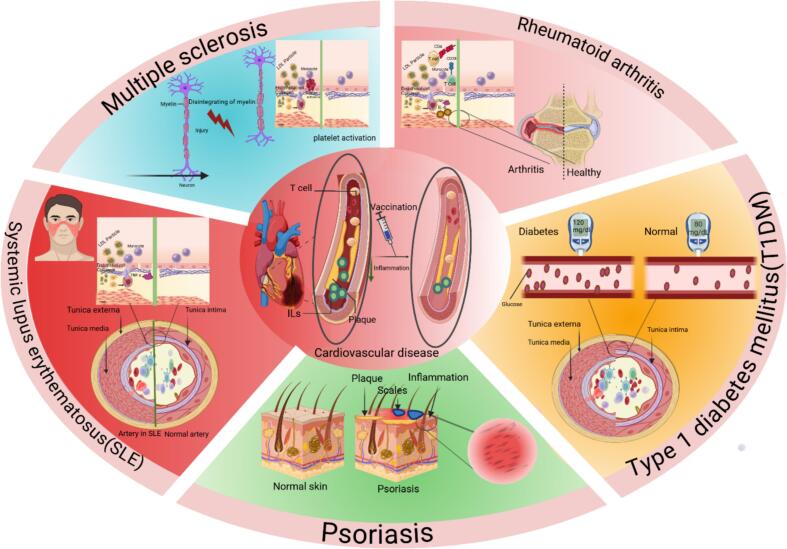
Autoimmune diseases with high risk for atherosclerosis. The vaccination against the progression of atherosclerosis is an essential approach to reduce the high death rate in autoimmune diseases. https://www.biorender.com

Autoimmune diseases such as systemic lupus erythematosus (SLE), rheumatoid arthritis (RA), multiple sclerosis (MS), and type 1 diabetes mellitus (T1DM) display a persistent systemic inflammatory environment that disrupts vascular homeostasis and accelerates atherosclerotic processes. This inflammatory state promotes endothelial activation and stimulates the expression of chemokines, adhesion molecules, and inflammatory genes, thereby increasing the risk of atherosclerosis [64] (Fig. 2).

### 2.1 Psoriasis

The chronic inflammation in psoriatic arthritis causes atherosclerosis to progress [65]. Boehncke et al. reported how inflammation develops atherosclerosis [66]. The studies also revealed that individuals with psoriasis have a higher likelihood of cardiovascular events and an elevated risk of heart attacks [67,68]. The patients with severe psoriasis also showed severe impairments in the function of micro-vessels in the coronary arteries [69]. Furthermore, the high arterial stiffness was directly related to the severity of psoriasis [70].

### 2.2 Multiple sclerosis (MS)

Cardiovascular diseases contribute significantly to the mortality of individuals with MS [71,72]. Vascular diseases may be more prevalent in individuals with MS through physiological processes, including platelet activation, endothelial dysfunction, hypercoagulation, and the use of drugs like glucocorticoids [73]. Some reports estimated that the incidence rates of stroke and heart failure are high among MS [74].

### 2.3 Systemic lupus erythematosus (SLE)

Cardiovascular diseases are believed to be an important factor in the death of SLE patients [75]. A high risk was estimated for coronary artery disease among SLE patients [64,76] since coronary artery calcification, carotid intima-media thickness, high blood ox-LDL, and cholesterol are reported in SLE [[77], [78], [79]]. The interferon α plays an important role in the development of SLE and triggers the absorption of lipids by macrophages, leading to the formation of foam cells and the development of plaques in the vessel sub-endothelial space [80].

### 2.4 Type 1 diabetes mellitus (T1DM)

T1DM Individuals showed a high risk for cardiovascular diseases [81]. The T1DM leads to the development of atherosclerosis, in which systemic inflammation plays a key role. The T1DM children have also shown high carotid intima-media thickness [[82], [83], [84]]. Notable changes in the speed of carotid-femoral pulse waves and dilation of blood vessels in response to endothelium-dependent flow have been observed in T1DM patients [81,85].

### 2.5 Rheumatoid arthritis (RA)

There is an increasing acknowledgment of the high death rate, primarily caused by atherosclerosis in patients with RA [86]. Autoimmune rheumatic conditions have been linked to lipid profiles, which can affect cardiovascular conditions [87]. The compounds that can degrade collagen play an essential role in disrupting atherosclerotic plaques in RA patients [64]. Some factors, such as IgM, TNF-α, and IL-6, are suggested to contribute to vascular diseases [88]. An increased number of CD4+ CD28null T cells, which invade the atherosclerotic plaques and display inflammatory events, can lead to vascular damage in RA patients [64].

## 3 Immunization is an approach against atherosclerosis

Many studies have reported the effectiveness of immunization to prevent the atherosclerosis process in animal models. Although atherosclerosis is not classified as a fully developed autoimmune disease, it shares many similarities with these diseases. It is also proposed that the prevention and treatment of atherosclerosis are feasible by employing similar methods for other autoimmune diseases [89]. Vaccination is suggested as the most effective preventative method for the majority of diseases associated with inflammation, making it a promising option for treating atherosclerosis [90]. The deletion of ADAMTS-7 by ATS7vac protects against both restenosis and atherosclerosis in mouse models [91]. Beyond plaque size reduction, most vaccines also shape adaptive immunity. For example, PCSK9-targeting peptide vaccines induced a dominant IgG1:IgG2a isotype switch, skewing toward Th2/Treg responses, which both neutralize PCSK9 and limit Th1-driven inflammation. Similarly, ox-LDL-pulsed DC vaccines expanded FoxP3^+^ Tregs, secreted IL-10, and suppressed effector T-cell proliferation. However, self-antigen vaccines carried a risk of epitope spreading: targeting B-cell epitopes on ApoB-100 may expose cryptic T-cell determinants, so rational epitope design is critical to preserve tolerance. The vaccination with the ApoB-100 peptide P210 also reduced the development of atherosclerosis in ApoE −/− mice [92,93]. The ANGPTL3 vaccine ameliorates dyslipidemia and minimizes the development of atherosclerosis in mice with familial hypercholesterolemia [94]. The vaccination using ox-LDL-pulsed mature DCs offers an effective approach for modulating atherosclerosis, decreasing the size of carotid artery lesions [95]. Furthermore, the MHC-II-restricted peptides derived from ApoB with incomplete and complete Freund's adjuvants were shown to protect mice against atherosclerosis [96]. L-IFPTA+ and AT04A vaccines reduced atherosclerotic lesions and protected mouse models against dyslipidemia by targeting PCSK9 [97,98]. Oral vaccination targeting TIE2 stimulates cellular immune response against cells with high TIE2 levels, reducing the size of atherosclerotic lesions [99]. Oral DNA vaccine targeting the murine VEGF receptor 2 (Flk-1) has been shown to have the potential to slow down the atherosclerosis process in mice [100]. Immunization of LDLR−/− mice with MDA-LDL and native LDL particles reduced atherosclerotic plaques [101,102]. The lesion was also reduced by vaccination targeting foam cells [103]. Administering the intranasal vaccine HB-ATV-8 controlled atherogenesis and fatty liver [104]. Immunotherapy methods on non-vaccine-based treatments were also reported in some studies. The G3BP2 immunotherapy reduced the atherosclerotic lesions in ApoE-deficient mice [105]. The plaque size at the aortic root of ApoE−/− mice decreased using oral HSP60 [106]. On the other hand, the use of Anti-BAFFR monoclonal antibody targeted mature B2 cells and disrupted spleen B cell zones but improved atherosclerosis in ApoE−/− mice [107].

## 4 Targeting of genes for immunization is based on their functions

**Fig. 3:**
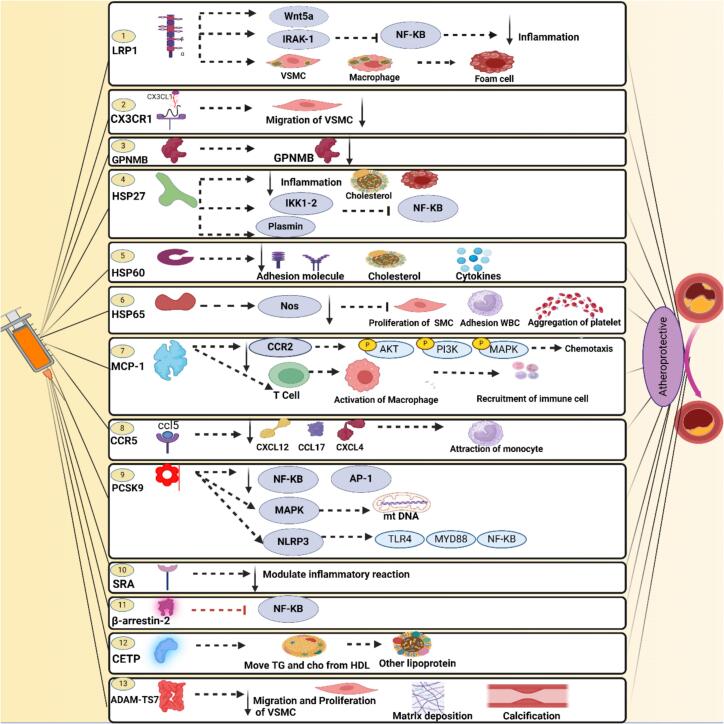
Gene targets for immunization against atherosclerosis. The genes are suggested to suppress the atherosclerosis process via different signaling axes. https://www.biorender.com

The inflammatory events keep some signaling axes and pathways active in the cells and help find the gene targets. For instance, the chemokine monocyte chemoattractant protein-1 (MCP-1 or CCL2) is highly expressed in activated endothelial cells and macrophages in response to TNF-α and IL-6 [46,47,88]. Studies have shown that MCP-1 levels are elevated in patients with SLE and RA, promoting the recruitment of monocytes, where they differentiate into foam cell-generating macrophages [64,88]. This is further supported by findings that link high serum IL-6 levels with increased coronary artery calcification and higher cardiovascular risk [[52], [53], [54]]. Likewise, CX3CL1 (fractalkine), a chemokine expressed by endothelial cells and vascular smooth muscle cells (VSMCs), and its receptor CX3CR1 are overexpressed in autoimmune contexts, facilitating leukocyte adhesion and transendothelial migration to develop atheromas [[64], [65], [66]]. These signaling axes are particularly relevant in the MS and SLE diseases, where immune-mediated vascular damage is a hallmark [71,77]. Moreover, genes involved in lipid metabolism, such as PCSK9, are dysregulated in autoimmune conditions. PCSK9 expression, primarily in hepatocytes and macrophages, is enhanced by inflammatory stimuli including TNF-α and IL-1β [50,64]. It exacerbates LDL receptor degradation, increases circulating LDL, and contributes to foam cell formation. The elevated PCSK9 levels have been observed in models of chronic inflammation relevant to autoimmune conditions [64,88]. The gene expression changes that occur under chronic inflammation create a pro-atherogenic state, characterized by immune cell infiltration, lipid dysregulation, and endothelial dysfunction, in autoimmune conditions. These findings support the hypothesis that targeting the genes in dysregulated pathways via vaccination may offer therapeutic benefits in autoimmune-related atherosclerosis. The control of these axes and pathways corrects and improves lipid metabolism, inflammation, and plaque formation in atherosclerosis. Here, some genes targeted in cellular axes and pathways (Fig. 3), and their roles are explained (Table 1).

**Table 1:** Gene targets.

Target Category	Gene(s)	Vaccine Type	Mechanism	Model	Reported Effect	Refs
Lipoprotein-Related antigens	PCSK9	Peptide/nanoliposome/VLP	↓LDL	Mouse, Rabbits	↓LDL, ↓plaque size	[97,98,156,174]
	ApoB-100 (e.g., P210)	Peptide oral/intramuscular	↑immune tolerance	ApoE^−/−^ mice	↓Lesion area	[92,93]
	CETP	Peptide/DNA (±HSP65)	↑HDL	Rabbits	↓Aortic plaque	[[194], [195], [196]]
Inflammatory mediators	IL-1β, IL-12, IL-17 A	Protein vaccines	↓cytokines	ApoE^−/−^ mice	↓Lesion burden	[50,55,56,60]
	CX3CR1/CCL2	DNA vaccine	↓chemotaxis	Mouse	↓Plaque area, no autoimmunity	[120,121]
Extra-and Intra cellular modulators	HSP60/65	Protein/DNA	↑IL-10, ↓IFN-γ	Rabbits	↓Plaque size, ↓inflammation	[128,129]
	ADAMTS-7	Peptide vaccine	↓Enzymatic activity	Mouse	↓Plaque size, ↑collagen	[89,91]
	CD99, TIE2	Protein/DNA	↓Endothelial activation	Mouse	↓Plaque growth	[99,199]

### 4.1 Lipoprotein receptor-related protein 1 (LRP1)

Lipoprotein receptor-related protein 1 (LRP1) is a multifunctional cell surface receptor involved in various physiological processes. It comprises two chains: the α chain (amino-terminal fragment) and the β chain (membrane-bound carboxyl fragment) [108]. LRP1 has emerged as a key player in atherosclerosis, influencing protective and detrimental pathways. This protein has an atheroprotective effect by modulating the Wnt5a signaling pathway, which is associated with cholesterol accumulation in fibroblasts [109,110]. LRP1 also interacts with tissue-type plasminogen activator and urokinase-type plasminogen activator [110]. Besides, its expression in VSMCs and macrophages, which affects the vasculature, is reported to be a protective factor against the development of atherosclerosis. On the other hand, the LRP1 can cause the accumulation of cholesteryl esters in VSMCs and macrophages, resulting in their transformation into foam cells and the development of atherosclerosis [110]. LRP1's β chain influences the expression of ATP-binding cassette transporter A1 (ABCA1) and neutral cholesterol ester hydrolase (NCEH1) [109,111,112]; and its α chain binds to Apo E, facilitating the interleukin-1 receptor-associated kinase-1 (IRAK-1) signaling pathway and reducing NF-κB-induced inflammation [113]. LRP1-targeted immunization in animal models has been shown to reduce lesion macrophages and plaque formation, potentially via the induction of anti-inflammatory regulatory T cells (Tregs) and modulation of cytokine profiles [97,109,113].

### 4.2 CX3CL1 (Fractalkine)/CX3CR1

CX3CL1 includes an extracellular domain linked to a stalk similar to that of mucin [114]. The mucin-like stalk is a unique feature attracting leukocytes that express CX3CR1 [115]. CX3CR1 is found on some cells, such as natural killer cells, T cells, smooth muscle cells, and monocytes, playing its role in their movement, attachment, and growth. Additionally, CX3CL1 is believed to support cell survival and functioning in normal and inflammatory circumstances [114]. It acts as a chemokine and adhesion molecule in monocytes [116]. CX3CL1 is also expressed throughout all phases of atherosclerosis in plaques found in the carotid artery [117]. Moreover, the CX3CR1 enhances the movement of smooth muscle cells during the later phases of atherosclerosis [118]. It has been demonstrated that CX3CL1 can damage the vascular endothelium while promoting cell survival and growth of blood vessels, ultimately impacting the stability and generation of the plaques [119]. DNA vectors carrying scFv for the DC-targeted receptor DEC205 were combined with the genes of CX3CR1 and CCL2 and administered weekly to C57/BL6 mice. All vaccinated mice produced antibodies and cellular responses against CX3CR1 and CCL2 within eight weeks. The serum containing these antibodies effectively inhibited the macrophage movement toward TNF-α/stimulated endothelial cells. These findings suggested that targeting self-antigens with DNA vaccines can generate functional immune responses that inhibit specific chemotactic agents, potentially offering a therapeutic strategy for atherosclerosis, where chemotaxis plays a crucial role in inflammation [120]. The impact of a DNA vaccine designed to target CX3CR1 showed that the anti-CX3CR1 antibodies are high without adverse reactions. Moreover, the vaccinated mice had smaller atherosclerotic plaques, with decreased lipid deposition and infiltration of macrophages in the brachiocephalic artery. The DNA/CX3CR1 vaccination showed a strong immune response and led to decreased atherosclerotic lesions accompanied by less infiltration of macrophages. It could be a therapeutic subject against chemokine pathways for atherosclerosis treatment [121]. Mechanistically, the antibodies generated by the DNA vaccine block chemokine-related signaling pathways, impairing monocyte recruitment to the endothelium. The inhibition of CCL2 (MCP-1) also reduces monocyte mobilization from the bone marrow and their entry into lesions [120]. As a result, vaccinated mice reduced plaque growth. In addition to neutralizing chemokine signaling, vaccination also induced Fc-dependent effector functions such as antibody-dependent cellular phagocytosis (ADCP) and modulated innate training and regulatory myeloid programming [120,121].

### 4.3 Glycoprotein non-metastatic melanoma protein B (GPNMB)

GPNMB, a protein spanning the cell membrane, is produced by macrophages and is identified in aortic plaques. GPNMB regulates lysosomal integrity and the lifespan of senescent cells [122]. Mice vaccinated against GPNMB reduced the number of GPNMB-positive cells. Age-related traits were improved, and the lifespan was prolonged by vaccinating against senescent cells. Although the reduction in plaque burden was associated with the clearance of senescent cells, the precise immune mechanisms remain undefined. Potential mechanisms may include the induction of antigen-specific cytotoxic T lymphocytes targeting senescent cells, modulation of immunosenescence-associated cytokine profiles, or unmasking of cryptic neoepitopes, which could carry a theoretical risk of epitope spreading [123].

### 4.4 Heat shock proteins (HSPs)

HSPs, known as molecular chaperones, are activated in response to various stresses. Their roles relate to transport, stability, protein folding, and inflammation [124]. HSPs' effects on atherosclerosis vary according to conditions such as intracellular versus extracellular localization, concentration, and intermolecular interactions. By shifting pro-inflammatory Th1 to anti-inflammatory Th2, HSP60 immunization reduced the production of cytokines and adhesion molecules. It also developed antibodies that bind to the surface of ox-LDL [125]. Furthermore, the vaccination with HSP65 had the potential to induce a Th1 polarized response, leading to the upregulation of NOS expression, so nitric oxide could stop the cell adhesion, the aggregation of platelets, and the proliferation of SMCs [126,127]. Zhong et al. reported a significant reduction of 33.6 % in plaque size at the aortic root during the early stages of atherosclerosis using the HSP60-containing vaccine [128]. In another study, rabbits receiving intranasal vaccines with plasmid DNA expressing HSP65 decreased cholesterol, IFN-γ, and elevated blood IL-10 [129]. Furthermore, the vaccination against HSP60 increased IL-10 and reduced IFN-γ levels in treated animals [129]. This immune deviation reduces endothelial activation and leukocyte adhesion. HSP immunization also triggers the production of antibodies that bind oxidized LDL on the plaque surface, facilitating clearance of ox-LDL and mitigating foam cell formation [125]. These results explain the reduced lesion size observed with HSP60/65 vaccines. Notably, isotype profiling of the anti-HSP antibody response in preclinical studies has demonstrated a dominance of IgG1 over IgG2a, suggesting a shift toward humoral Th2-type immunity. However, the implications for immune memory and tolerance durability remain untested [125,128,129].

HSP27 has been shown to improve atherosclerotic plaque stability by modulating plasmin and other extracellular potential mediators of VSMC apoptosis [130,131]. Extracellular HSP27 may protect the endothelium from stress in the vasculature. It also acts as an antioxidant during oxidative stress, lowering ROS levels in endothelial cells and protecting the endothelium from ischemic insult by maintaining the integrity of cytoskeletal proteins and acting as an anti-apoptotic protein. HSP27-based treatment strategies may reduce lesion formation and improve re-endothelialization after vascular injury. Laboratory results suggested that extracellular HSP27 may also play a role in the regeneration of the endothelial barrier [132]. Clinically, low circulating HSP27 levels have been associated with advanced atherosclerosis and adverse cardiovascular events [133,134]. Histological studies localize HSP27 to the fibrous cap and media of atheromas, whereas its level is lower in the necrotic core of plaques, possibly due to proteolytic degradation [[135], [136], [137]]. Notably, the highest HSP27 expression is observed in areas adjacent to plaques, coinciding with active inflammation. In experimental models, overexpression of HSP27 reduces serum cholesterol levels and attenuates foam cell formation, implying a role in lipid homeostasis. Mechanistically, HSP27 competes with modified LDL for the scavenger receptor-A on macrophages, thereby reducing foam cell development. HSP27 also promotes cholesterol efflux by enhancing the activity of ABCA1 and ABCG1 transporters [138,139]. Furthermore, HSP27 can modulate inflammatory signaling pathways by binding to IKK and inhibiting NF-κB activation in macrophages, which helps stabilize plaques by reducing inflammatory genes [140,141]. Given its protective properties against atherosclerosis, HSP27 has emerged as a possible therapeutic target for cardiovascular disease. Strategies for increasing HSP27 in atherosclerotic lesions may help to stabilize susceptible plaques and minimize the risk of plaque rupture and thrombosis [142,143]. Thus, focusing on various HSP subtypes may be a novel and useful strategy for treating or preventing atherosclerosis by influencing the inflammatory and immunological responses in the arterial wall.

### 4.5 Monocyte chemoattractant protein-1 (MCP-1/CCL2)

Monocyte chemoattractant protein-1 (MCP-1/CCL2) is a key chemokine driving chronic arterial inflammation. Endothelial cells and macrophages secrete MCP-1 in response to inflammatory stimuli (e.g., oxidized LDL). MCP-1 then binds to its receptor (CCR2) on circulating monocytes and T cells, triggering PI3K/Akt/MAPK signaling axis that induces cytoskeletal changes and chemotaxis [144,145]. This process promotes the recruitment of immune cells through the arterial wall, where monocytes differentiate into macrophages and take up ox-LDL to form foam cells. Other chemokines (e.g., CCL5/RANTES) similarly recruit leukocytes and have been linked to plaque progression and instability [146,147]. Studies also reported that the MCP-1-mediated recruitment of T cells exacerbated the inflammatory responses in atherosclerosis. The T cells release cytokines and chemokines, which activate the endothelial cells and macrophages, resulting in greater recruitment of immune cells in the atherosclerotic lesions [148]. Moreover, the atherosclerotic plaques promote a pro-inflammatory milieu, including the release of MCP-1, cytokines, and matrix metalloproteinase. These factors cause plaque instability, which can lead to rupture and thrombosis, resulting in acute cardiovascular events like myocardial infarction or stroke [149]. Also, VSMCs with proliferative phenotypes are one of the most common cell types in lesion sites. Some studies revealed that the MCP-1 enhances α-SMA content in aortic root plaques [144]. The various signaling pathways involved in MCP-1-mediated inflammation during the atherosclerosis process help identify possible treatment targets. Targeting critical components of the MCP-1 signaling cascade may provide new approaches for controlling immune cell recruitment and inflammation in atherosclerotic plaques [150,151]. In vaccine models, targeting MCP-1 or CCR2 has led to a decrease in monocyte infiltration and lesion macrophages, potentially involving downstream effects on T cell activation, effector cytokine profiles, and even tolerogenic reprogramming of myeloid precursors [120,121,148]. Clinically, elevated MCP-1 levels are correlated with the severity and outcomes of atherosclerosis. For example, the high circulating MCP-1 level is associated with increased carotid plaque volume and vulnerability [152], as well as higher risks of coronary artery disease and stroke in genetic and cohort studies [153,154]. Likewise, tissue studies reveal higher MCP-1 levels in unstable plaque regions, supporting its role in plaque destabilization [155]. Thus, MCP-1/CCR2 signaling axis is a potential therapeutic target for controlling immune-cell recruitment in atherosclerotic lesions.

### 4.6 Proprotein convertase subtilisin/kexin type 9 (PCSK9)

PCSK9 plays an important role in the pathogenesis of atherosclerosis. It is expressed by hepatocytes, macrophages, and vascular smooth muscle cells [[156], [157], [158]]. The PCSK9's primary function is to regulate cholesterol homeostasis by modulating the LDLR. It binds to LDLR, leading to their internalization and lysosomal degradation, and prevents the recycling of LDLR to the cell surface, so that the LDL particles increase in circulation and cause hyperlipidemia, a key factor in developing atherosclerosis plaque. Furthermore, PCSK9 is affected by lipid, oxidative stress, and inflammatory cytokines. In an inflammatory milieu, PCSK9 contributes to foam cell formation, a hallmark of early atherogenesis, by enhancing the scavenger receptors, which in turn promote the ox-LDL uptake. Simultaneously, PCSK9 inhibits the efflux of cholesterol from macrophages, resulting in the conversion of macrophages into foam cells [159,160]. Furthermore, the PCSK9 induces the production of inflammatory cytokines, adhesion molecules, and chemoattractants within the vascular wall. PCSK9 also activates macrophage proinflammatory effects by TLR4. It triggers the NF-кB activation and nuclear translocation. It also stimulates the expression and secretion of various proinflammatory cytokines, contributing to increased monocyte infiltration and plaque deposition [161,162]. The PCSK9 can promote a phenotypic switch in vascular smooth muscle cells from a contractile to a synthetic state that induces cell proliferation and extracellular matrix production as a key event in plaque progression. In VSMCs, the PCSK9 is enhanced by mtDNA damage via the upregulation of p-mTOR. In turn, the PCSK9 increases mtDNA damage by regulating the apoptotic proteins via the MAPK signaling pathway. Besides, low shear stress increases the PCSK9, which upregulates AP-1 and NF-κB [[160], [161], [162], [163]]. The ATP and nigericin simultaneously induce the NLRP3 inflammasome and PCSK9. Hypoxia can also induce PCSK9 and activate the NLRP3 inflammasome. The NLRP3 inflammasome, containing NLRP3, ASC, and pro-caspase-1, also regulates the secretion of PCSK9, while MAPKs play a key role in regulating the IL-1β-mediated PCSK9 secretion. PCSK9 can directly activate the NLRP3 inflammasome and promote the secretion of pro-inflammatory cytokines via NF-κB. Furthermore, PCSK9 damages mtDNA and subsequently induces pyroptosis. Another mechanism underlying the effects of PCSK9 on NLRP3 inflammasome activation involves TLR4/MyD88/NF-κB signaling axis [[164], [165], [166]]. The activation of the TRIF/NF-κB and TLR4/MyD88 leads to the formation of the NLRP3 inflammasome. The NLRP3 induces caspase-1, facilitating the cleavage of pro-IL-1β and pro-IL-18. Additionally, the activated caspase-1 cleaves GSDMD and generates an active GSDMD-NT, which translocates to the plasma membrane and forms a pore that allows the secretion of proinflammatory cytokines like IL-1β and IL-18 [167,168]. The therapeutic approaches targeting PCSK9 have been developed due to the effects of PCSK9 on atherosclerosis. The monoclonal antibodies and small interfering RNA (siRNA) therapeutics have been notably effective in reducing LDL-C levels and mitigating cardiovascular risk. These therapeutic approaches have presented novel opportunities for addressing hypercholesterolemia and mitigating the risk of atherosclerosis [97,169,170]. The PCSK9-specific antibodies that disrupt the interaction of PCSK9 with LDLR have been evaluated successfully in studies [97,142,[170], [171], [172], [173], [174]]. Galabova et al. employed a conjugated version of short peptides (8–13 amino acids) from the human PCSK9 protein's N-terminal region. Keyhole limpet hemocyanin (KLH), a foreign carrier protein, is conveyed in this conjugated form, which contains non-self T cell epitopes [156]. They showed the induction of PCSK9 antibodies and the decrease of total cholesterol in Wistar rats. Six months after their initial vaccination, mice carrying the Balb/c mutation still had lower total cholesterol levels than the control groups [175]. PCSK9 peptide immunization reduced plasma levels of PCSK9 and total cholesterol, along with atherosclerotic lesions and vascular inflammation [98,176]. Non-self-epitopes were developed using AFFITOME technology. While these peptides are different from the original PCSK9 peptides, they share certain characteristics [177]. Crossey et al. used the Qβ bacteriophage virus-like particle (Qβ-VLP) vaccine platform to treat atherosclerosis [178]. Based on the known structures of LDLR and PCSK9, five regions of PCSK9 were identified as candidates for the binding of LDLR [158]. These findings suggested that by lowering atherogenic LDL-C and regaining the physiological function of LDLR, the PCSK9 vaccination is promising for treating or preventing atherosclerosis. Mechanistically, PCSK9 antibodies prevent PCSK9 from binding the LDL receptor. This spares LDLR from degradation, leading to increased clearance of LDL from the bloodstream and thus lower plasma cholesterol [156]. In addition, by reducing PCSK9 activity, such vaccines indirectly diminish PCSK9's pro-inflammatory effects in vascular cells. Moreover, peptide-based PCSK9 vaccines in murine models have been associated with class switching toward IgG1-dominant responses, indicating a humoral Th2 bias, and in some protocols, expansion of antigen-specific Tregs, though the durability of tolerance and risks of cross-reactive epitope spreading remain incompletely defined. Interestingly, co-targeting PCSK9 and IL-1β (e.g., dual vaccination or combination with canakinumab) may synergistically suppress both lipid and inflammatory drivers of atherosclerosis [97,98,174].

### 4.7 Scavenger receptor A (SRA/CD204)

SRA, also known as CD204 or macrophage scavenger receptor (MSR), binds to different ligands [179]. It is predominantly detected in immune cells, specifically in the innate immune system, including DC and macrophages. The SRA can bind various ligands, including bacterial components, modified substances, and endogenous stress proteins. Some findings suggested that the SRA/CD204 has the potential to modulate inflammatory reactions [180]. Many studies have also demonstrated that the lack of SRA/CD204 reduced the defense against pathogen invasion, potentially due to heightened vulnerability to excessive proinflammatory cytokine production [180,181]. Yi et al. reported a stronger CD4^+^ T cell reaction following ovalbumin immunization in mice lacking SRA/CD204. Their results also demonstrated the role of SRA/CD204 in limiting the natural immunogenicity of antigen-presenting cells. In the context of vaccination, the suppression of antigen presentation via SRA/CD204 restrained effective T cell priming and skewed cytokine polarization, which in turn affects both effector and regulatory responses. Antigen-specific immune responses could be modulated by altering the threshold of dendritic cell activation or altering peptide-MHC density. However, the full downstream consequences for memory generation, isotype commitment, or regulatory polarization have not yet been delineated [182].

### 4.8 Beta arrestin (β-arrestin)

β-arrestin 1 and 2, traditionally known for desensitizing GPCR signaling, have divergent effects on cardiovascular disease. β-arrestin1 has been linked to adverse cardiac remodeling, whereas β-arrestin2 exhibits cardio-protective effects, including dampening inflammation and apoptosis, as well as improving post-MI cardiac function. Mechanistically, β-arrestin2 can inhibit NF-κB activation in cardiomyocytes and enhance SERCA2a activity, thereby improving contractility [183]. Emerging evidence indicates β-arrestin2 may have context-dependent roles in atherosclerosis. Paradoxically, ApoE−/− knockout mice lacking β-arrestin2 develop significantly less atherosclerosis, suggesting that β-arrestin2 can promote plaque formation under certain conditions. This finding raises the prospect that inhibiting β-arrestin2 activity could be a therapeutic strategy to combat atherosclerosis and post-angioplasty restenosis [184]. Supporting this, angiotensin II through its AT1 receptor has a β-arrestin-dependent arm, resulting in β-arrestin2 being found to be doubled in atherosclerotic human coronary arteries [185,186]. Conversely, other data indicate β-arrestin2 deficiency can enhance dendritic cell migration and cytokine production, underscoring the complex influence of β-arrestin2 on vascular inflammation [187]. β-arrestin also acts as a signaling hub, linking cellular processes like the mitogen-activated protein kinase (MAPK) pathway, DNA replication, protein synthesis, and cell migration. It was also reported that the β-arrestin2 triggers a chain reaction involving c-Raf1, MEK1, and ERK1/2 [188]. These findings highlighted how these crucial pathways cooperate to regulate cellular behavior [189]. In preclinical models, β-arrestin-targeted vaccination strategies remain exploratory, but modulation of DC trafficking and antigen presentation via β-arrestin2 signaling has been proposed to influence T cell priming thresholds and peripheral immune tolerance, especially within inflamed vascular beds [184,187].

### 4.9 Cholesteryl ester transfer protein (CETP)

CETP is a plasma protein that transfers triglycerides and cholesteryl esters from HDL to other lipoproteins. Blocking CETP could be a potential choice against atherosclerosis. The capacity of vaccines to alter the CETP function in atherosclerotic animal models has been the subject of substantial investigation [190]. Although controversial results, some studies have demonstrated that the CETP-specific antibodies decrease the CETP activity [[191], [192], [193]]. The HDL-cholesterol (HDL—C) levels increased in rabbits immunized against CETP-induced neutralizing antibodies, which is linked to a reduction in the atherosclerosis process [194]. Additionally, a vaccine that targets both CETP and HSP65 nasally has been shown to lower the incidence of aortic atherosclerosis in rabbits [[195], [196], [197]]. On the other hand, Torcetrapib, the first small-molecule inhibitor of CETP, showed no regressions of coronary atherosclerosis [198]. These reports suggested that the CETP vaccination could be a promising way to prevent the progression of atherosclerosis. However, there is still a long way to go before it can be truly evaluated. In experimental models, anti-CETP immunization has been shown to induce class-switched IgG antibodies capable of complement activation and Fc receptor engagement, which may contribute to enhanced CETP clearance and modulate macrophage lipid handling and inflammatory tone [191,194,196].

### 4.10 ADAMTS-7

Many cells, such as macrophages, SMCs, and endothelial cells, express ADAMTS-7 metalloprotease. This enzyme is involved in the context-dependent remodeling and disintegration of the extracellular matrix (ECM)**,** particularly in atherosclerosis. It catalyzes substrates such as aggrecan, thrombospondin-1, and cartilage oligomeric matrix protein (COMP), impacting processes like cell adhesion, migration, proliferation, and ECM calcification. Genome-wide association studies showed that the ADAMTS-7 gene locus relates to human coronary atherosclerosis, underscoring its importance in vascular pathology. Several studies have investigated the possibility of neutralizing the enzyme activity using ADAMTS-7-specific antibodies, primarily in preclinical models. The findings demonstrated that the ADAMTS-7 vaccination can considerably reduce plasma ADAMTS-7 levels, aortic plaque size, and inflammation. This immunization works by inducing an immune response against ADAMTS-7, thereby reducing its activity. Furthermore, the ADAMTS-7 immunization can potentially affect the expression of other genes and proteins that control inflammation, matrix homeostasis, and lipid metabolism. For example, it may influence inflammation-related pathways and extracellular matrix stability**.** These reports suggested that by blocking ADAMTS-7's proteolytic activity and preserving the integrity and stability of the arterial wall, ADAMTS-7 immunization might be a unique and successful way to treat and prevent atherosclerosis [89,91]. By generating antibodies against ADAMTS-7, the vaccine blocks ADAMTS-7's enzymatic degradation of matrix components in the arterial wall. This preservation of the extracellular matrix maintains plaque stability and may reduce VSMC migration into lesions [91]. Additionally, neutralizing ADAMTS-7 appears to downregulate local inflammatory signals, thereby attenuating plaque inflammation [89,91]. It is plausible that ADAMTS-7-targeted immunization may engage Fc-mediated clearance mechanisms, alter antigen uptake dynamics by antigen-presenting cells, or shape local lymphoid microenvironments that influence the balance between effector T cell activation and tissue-specific regulatory circuits [89,91].

### 4.11 Other potent gene targets

Multiple studies reported that some genes, such as CD99, HB-ATV-8, miR-203-3p, GTPCH1, Cx37, cytokines, and CX3CR1, may be amenable to gene therapy and vaccination. Immunizing against CD99 can decrease plaque formation and the movement of monocytes and T cells in the arterial wall of LDLR-deficient animals [199]. An animal study on atherosclerosis showed that intranasal administration of the synthetic peptide HB-ATV-8 reduces the plasma cholesterol and triglyceride levels, induces a Th2 immune response, protects against non-alcoholic fatty liver disease, and atherosclerosis [104]. Macrophages can reduce atherosclerosis by transferring exosomal miR-203-3p from DCs. It inhibits the expression of cathepsin S, a protease that degrades LDLR [200]. Inducing the production of vascular GTPCH1 and endogenous tetrahydrobiopterin (THB) can enhance nitric oxide levels and reduce oxidative stress [201]. A specific DNA vaccine targeting CX3CR1 inhibits atherogenesis in mice by decreasing the accumulation of CX3CR1-expressing cells in the plaque [121]. The prevenar-13 vaccine induced PC- and ox-LDL-specific IgM and IgG in individuals who received two separate six-month doses [202]. Future studies are needed to determine the most reliable panel of markers to use in clinical practice in the patient population [203]. Other genes involved in metabolic and cardiovascular diseases might also be targets for therapeutic oligonucleotides, such as microRNAs, small interfering RNAs, and antisense oligonucleotides. These reports suggested that a new and effective approach to the prevention and treatment of atherosclerosis could be to change the molecular pathways underlying the disease [204].

As detailed in Table 1, candidate vaccines fall into three primary categories: those targeting lipoprotein-related antigens (e.g., PCSK9, ApoB-100, CETP), inflammatory mediators (e.g., IL-1β, IL-12, IL-17 A, CX3CL1/CCL2), and other extracellular and cellular modulators (e.g., HSPs, ADAMTS-7, CD99). Lipoprotein-targeted vaccines, such as peptide-based PCSK9, consistently reduced cholesterol levels and plaque burden in animal models [92,93,97,98,156,174]. The CETP and ApoB-100-based vaccines enhanced HDL or induced antigen-specific tolerance, respectively, contributing to lesion attenuation [92,93,[194], [195], [196]]. Inflammatory cytokine and chemokine-directed vaccines, such as those neutralizing IL-12 or blocking CX3CR1/CCL2 interactions, effectively suppressed monocyte recruitment and dampened vascular inflammation in preclinical studies [50,55,56,60,120,121]. Finally, structural and immune modulator-based targets, including HSPs and ADAMTS-7, demonstrated promising outcomes in restoring plaque stability, reducing inflammatory cell infiltration, and preserving extracellular matrix architecture [89,91,99,128,129,199]. Collectively, these strategies underline a paradigm shift from passive lipid-lowering to active immunological modulation of atherosclerosis. Although still under preclinical or early-phase evaluation, these vaccine candidates illuminate multiple entry points for antigen-specific atheroprotection.

## 5 Immunization strategies and approaches

### 5.1 Biological basis

The vaccines studied experimentally in atherosclerosis include DNA-based vaccines, peptide vaccines, and mRNA vaccines [205]. These vaccines are designed to target specific antigens or pathways that contribute to the development of atherosclerosis [206]. DNA-based vaccines deliver genetic materials encoding specific antigens related to atherosclerosis, while peptide vaccines consist of short protein fragments derived from atherosclerosis-associated molecules. The mRNA vaccines, on the other hand, use the RNA to produce antigens that trigger the immune responses against atherosclerosis-related targets [207]. Understanding the potential effectiveness of these vaccines in preventing or treating atherosclerosis requires knowing their nature and how they interact with the immune system [208].

### 5.2 Effectiveness

The vaccines have been reported to diminish the progression of atherosclerosis by the immune responses targeting key molecules like ox-LDL and genes of inflammatory pathways in animal models [209]. Studies highlighted the potential of vaccination to address the underlying mechanisms of atherosclerosis [210]. Although animal studies yielded promising results, the effectiveness of these vaccines in humans remains to be investigated in clinical trials [[211], [212], [213]]. However, imaging modalities have expanded for coronary arteries, but these tools have limitations that hinder their clinical applicability. For instance, coronary angiography, the most commonly used technique, focuses solely on imaging the vessel lumen but cannot assess vascular characteristics comprehensively [214,215]. It is suggested to apply the therapeutic tools directed at atherosclerotic plaques for monitoring atherosclerotic lesions [216]. These strategies offered the potential for localizing treatment, reducing systemic side effects, and optimizing therapeutic efficacy, especially when utilizing novel anti-inflammatory agents [217]. The development of such targeted therapies holds promise for enhancing clinical outcomes and addressing concerns related to treatment costs and systemic susceptibility to infections in atherosclerosis management [218]. However, the vaccine's effectiveness and stability depend on some factors, such as the vaccine's type, formulation, and administration method [219,220]. Long-term studies are needed to evaluate the persistence of immune memory, the ability of vaccines, and immune responses [221,222].

### 5.3 Preclinical efficacy

The preclinical efficacy relates to vaccination results obtained from animal models. For example, immunization against PCSK9 consistently led to lowered total cholesterol (≈30–50 % reduction) and smaller aortic lesions in mice [98]. Peptide vaccines targeting ApoB-100 (the P210 peptide) yielded ∼60 % reductions in plaque area in ApoE-deficient mice compared to controls [92,93]. Vaccines targeting pro-inflammatory cytokines such as IL-12 have demonstrated atheroprotective effects, with treated mice exhibiting reduced lesion size and dampened vascular inflammation compared to controls [60]. To date, no atherosclerosis vaccine has been used; the effectiveness in humans remains to be determined in future trials [[211], [212], [213]]. Torcetrapib's Phase III failure due to off-target aldosterone upregulation and unexpected cardiovascular events caused it to falter in humans. Some early-phase human studies are underway, but their outcomes have not yet been published. Overall, the animal data support the concept that active immunization can mitigate atherosclerosis, but translation to human therapy will require careful evaluation. The divide between preclinical promise and clinical stagnation stems from more than species differences. Table 2 outlines a comprehensive framework of translational gaps, ranging from immunologic oversimplification to endpoint mismatches and feasibility limits.

**Table 2:** Challenges on immunization among preclinical models.

Challenge Domain	Preclinical Strength	Clinical Barrier	Contrast Summary	Examples/Refs
Immune Modulation	Controlled Treg/Th1/Th2 skewing via defined antigens in genetically identical mice	Human immune responses are polyclonal, variable, and affected by age, infections, and comorbidities.	Predictable vs. chaotic immunology	PCSK9 [97,98], ApoB [92,93], ox-LDL-DCs [95]
Lesion Size Reduction	Plaque area measured by en face staining or histology → 20–70 % reduction	Human plaque rupture risk depends on fibrous cap thickness, calcification, and necrotic core, not just size.	Morphometry vs. clinical vulnerability	ApoB vaccines [92,93], IL-1β [60]
Timing of Intervention	Vaccines given before plaque development (prophylactic) in young mice	In real patients, plaques are already established at the time of intervention	Early prevention vs. late salvage	HSP60 [128,129], PCSK9 [174]
Antigen Dose & Schedule	High antigen/adjuvant dose with prime-boost every 1–2 weeks	Limited vaccine repetition in humans due to cost, safety, and compliance	Flexible murine dosing vs. strict human feasibility	P210 [93], CETP vaccines [[194], [195], [196], [197], [198]]
Model Uniformity	ApoE^−^/^−^ mice on high-fat diet → predictable plaque formation in all animals	Human populations vary in HLA alleles, lipoprotein subclasses, gut microbiota, and drug history.	Genetic clone vs. real patient variability	AT04A [97,98], HB-ATV-8 [104]
Outcome Measurement	Clear endpoints: plaque %, IgG titers, IL-10/IFN-γ ratios	Human trials must rely on surrogates (LDL, hsCRP) or hard endpoints (MI, stroke) with long timelines	Mechanistic clarity vs. clinical ambiguity	CANTOS [60], AT04A [98]
Autoimmunity Surveillance	Few signs in mouse models due to short follow-up, immune privilege	In humans, T-cell activation can emerge years later (e.g., epitope spreading, TCR escape)	Immunologic latency is ignored in models	ApoB100 [92,93], ox-LDL [95]
Mechanism Clarity	Mechanism often inferred from knockout models or cytokine readouts	In humans, causal inference is weak; often, correlation without mechanistic clarity	Gene-targeted certainty vs. human causal fog	IL-1β [[50], [51], [52], [53], [54], [55], [56], [57], [58], [59], [60]], PCSK9 [174]
Mucosal Vaccines	Intranasal/oral delivery works in mice with a strong local IgA/IL-10 response	Human mucosa has variable IgA levels, microbiome interference, and mucin barrier thickness.	Clean mucosa vs. human unpredictability	HSP65 [128], HB-ATV-8 [104]

### 5.4 Side effects

The adverse effects reported are generally mild, including transient local inflammation at the injection sites and temporary elevation of cytokines, typical of other immunization platforms [223,224]. For example, vaccines targeting cytokines such as IL-1β or lipid-related molecules like PCSK9 and oxidized LDL have shown favorable safety profiles in animal studies, without evidence of systemic autoimmunity or tissue damage [225,226]. Nevertheless, these vaccines often target self-antigens, such as endogenous lipoproteins and stress proteins like HSP60, which raises theoretical risks of autoimmunity. A critical concern involves the potential for molecular mimicry or epitope spreading, particularly in vaccines that induce immunity against oxidized LDL. Thus far, long-term surveillance in animal models has not indicated serious adverse outcomes, but these theoretical risks warrant caution. Reports from peptide immunotherapies in chronic inflammatory settings suggest that rational antigen design—avoiding T-cell epitopes that trigger loss of tolerance—may reduce unintended immune activation. Any vaccine candidate advancing to clinical trials will be subject to strict regulatory oversight. According to vaccine development protocols, such candidates must undergo phased monitoring for delayed-onset adverse events, including immune complex deposition or autoreactive flares [227,228]. In conclusion, although current data support a favorable preclinical safety profile, translational progress must proceed with robust immunological monitoring to ensure safety in human applications.

### 5.5 Delivery

In order to improve delivery, many studies reported the use of nanoparticles and nanocarriers in the production of vaccines against atherosclerosis. These nano constructs also showed other beneficial characteristics, such as the prevention of enzymatic degradation [93,229], targeting antibodies [230], and elevation of epitope density [93] (Table 3). Despite these delivery advances, critical translational hurdles persist such as target specificity and autoimmunity (vaccines against self-antigens (e.g., PCSK9, HSP60, ox-LDL) risk molecular mimicry, potentially triggering immune responses in humans despite preclinical safety), inflammation exacerbation (Adjuvants or Th1-skewed responses (e.g., HSP65-induced IFN-γ) may paradoxically amplify vascular inflammation, accelerating plaque vulnerability.) clinical evidence gaps (animal models poorly replicate human plaque), complexity, comorbidities (e.g., diabetes), and immune aging.

**Table 3:** Gene nanoparticles and nanocarriers.

Vaccine/ Particle/Carrier	Animal	Function	Authors	Year
Chitosan/pCETP nanoparticles	Rabbit	Decrease of pCETP and the aortic lesion area	Xiying Yuan, et al. [229]	2008
PCSK9 Hapten multi-copy displayed onto carrier protein nanoparticle (PMCDN)	Mouse	Increase of antibody	Shasha You, et al. [173]	2019
PCSK9 peptide anchored on the surface of hybrid nanocarriers	Mouse	Increase of antibody against PCSK9PCSK9	Haiying Ji, et al. [230]	2020
Vesicular nanocarrier for co-delivery of P210 peptide and 1, 25-Dihydroxyvitamin D3	Mouse	Decrease of vascular lesion area, vascular stiffness, and macrophage content.	Sijia Yi, et al. [231]	2019
Liposomal Immunogenic Fused PCSK9-Tetanus plus Alum adjuvant (L-IFPTA)	monkey	Increase of antibody against PCSK9	Amir Abbas Momtazi-Borojeni, et al. [174]	2021
ApoB-derived P210 peptides/retinoic acid/ PLGA nanoparticles	Mouse	Decrease of cholesterol and the aortic lesion area	Xianwen Yi, et al. [232]	2020
Intranasal vaccine HB-ATV-8	Pig	Increase of anti-CETP. Decrease of the lipid droplets, foam cells, and VSMCs	Roxana Gutierrez-Vidal, et al. [104]	2018
Delivering P210 peptide nanoparticles	Mouse	Decrease of CD4^+^ effector memory T cells, Splenic IL-1 receptor type 1 (IL-1R1), IL-6, and IL-17a. Increase of CD8^+^ central memory T cells, CD4^+^CD25^+^FoxP3^+^ regulatory T cells, and CD8^+^CTLA-4^+^ T cells.	Kuang-Yuh Chyu, et al. [93]	2022
Immunogenic fused PCSK9-tetanus (IFPT) on the liposome nanoparticles (L-IFPT)	Mouse	Increase of antibody against PCSK9	Amir Abbas Momtazi-Borojeni, et al. [97]	2021
ADAMTS-7 derived Epitopic peptides Carrier	Mouse	Decrease of the intimal thickening, neointima formation, degrees of atherosclerotic lesions, and VSMC migration.	Zihan Ma, et al. [91]	2023
Apolipoprotein(a) Carrier	Mouse	Increase of anti-apo(a). Decrease of media thickness and macrophage migration.	Mariko Kyutoku, et al. [233]	2013
DNA/CETP epitope Carrier	Rabbit	Increase of anti-CETP. Decrease the aortic lesions and the vessel thickness.	Dan Mao, et al. [234]	2006
Anionic phospholipid 1,2-distearoyl-sn-glycero-3-phosphoglycerol (DSPG) Carrier	Mouse	Decrease of plaque formation.	Naomi Benne, et al. [235]	2018
Cholesterol and lipid A Carrier (as an adjuvant)	Rabbit	Increase of anti-cholesterol. Decrease of the risk of atherosclerosis.	Carl R. Alving, et al. [236]	1996
Tetanus Toxoid-CETP Carrier	Rabbit	Increase of anti-TT-CETP. Decrease of the vessel thickness.	Tamara Aghebati, et al. [237]	2020
CETP and p210 epitope Carrier	Mouse	Increase of anti-CETP and anti-p210	Dania O. Govea-Alonso, et al. [238]	2017
ApoB-100/p210 Carrier	Mouse	Increase anti-p210.	Josue´ I. Beltra'n-Lo'pez, et al. [239]	2016
DNA/CETP Carrier	Rabbit	Increase in anti-CETP.	Xiaorong Yang, et al. [240]	2009
DNA/targeted TIE2 Carrier	Mouse	Decrease of the TIE^+^ cells, carotid atherosclerosis, intima area, and intima/lumen ratios.	Arnaud D. Hauer,et al. [99]	2009
HSP65 Carrier	Mouse	Increase in anti-HSP65. Decrease in atherosclerotic lesion formation and endothelium damage.	Hou Jing, et al. [241]	2011
CETP epitope Carrier	Mouse	Increase anti-CETP.	Qi Gaofu,et al. [242]	2004
tri-peptide construct (AHC) Carrier	Mouse	Decrease of lipids and macrophage infiltration. Increase of inflammatory mediators.	Vrushali eshpande, et al. [243]	2016
Tetanus Toxoid-CETP Carrier	Rat	Increase anti-CETP.	Qi Gaofu, et al. [244]	2005

## 6 Future of immunization against atherosclerosis

Vaccination against specific atherosclerosis-related antigens could potentially mitigate plaque development by eliciting protective immune responses. Early studies in this field focused on identifying suitable antigens (ox-LDL, ApoB-100, CETP, PCSK9, HSP60, MHC-II peptides, interleukins) and appropriate adjuvants to induce immunity [89,90]. DNA-based vaccination has opened new doors in this field; however, an ideal vaccination that can be used to protect people from atherosclerosis is still far from being developed [209]. The practical and effective improvement of techniques in vaccination, selection of formulation, route of administration, vaccine safety and stability, timing and duration of vaccination, appropriate definition and monitoring of efficacy endpoints, and potential side effects of vaccination, such as undesired immune activation, all of which will need to be addressed in future clinical trials. Immunotherapy offers advantages over conventional treatments (fewer off-target effects, more durable responses, higher efficacy). However, its use also has limitations. In practice, relatively high doses of immunomodulators are often required chronically, which can lead to autoimmune side effects. Biological therapies may also suffer from enzymatic degradation, poor tissue penetration, and rapid clearance by the reticuloendothelial system. Drug delivery strategies (DDSs) refer to platforms like liposomes or PLGA nanoparticles designed to passively deliver antigens or adjuvants, enhancing bioavailability and tissue targeting. These biomaterial approaches to drug delivery can enhance the delivery of biological materials to their intended targets. In preclinical studies, DDSs have demonstrated superior and remarkable efficacy in immunotherapy for various inflammatory diseases. In light of its excellent safety and promising industrial prospects, DDSs could be a viable option for immunotherapy. DDSs include liposomes, liposome-like NPs, degradable polymeric carriers such as PLGA NPs and microspheres, albumin-based NPs, and cellular carriers such as red blood cells [245]. New therapeutic strategies in atherosclerosis involve understanding the immune agents in processes such as leukocyte adhesion, cellular migration, and progression of plaques. Targeted therapeutic approaches that modulate these pathways could offer significant advancements in treating atherosclerosis [246]. It is well known that the inflammatory cells involved in the arterial wall can secrete cytokines such as TNFα, IL-1β, and IL-6, which contribute to plaque growth. The successful development of monoclonal antibody-based immunotherapies targeting specific cytokines in the treatment of chronic immunoinflammatory diseases associated with high cardiovascular risk, such as psoriasis, RA, and ankylosing spondylitis, offers an exciting opportunity to fill this knowledge gap [247]. Many therapeutic policies in atherosclerosis cause severe immunosuppression and an increased risk of arterial thromboembolic events. A recent report has suggested utilizing the therapeutic potential of the CD40-CD40L. Antisense oligonucleotides (ASOs) and siRNAs are also being investigated to target CD40. SiRNA-mediated CD40 inhibition in mice reduced atherosclerotic lesions through the NF-κB signaling pathway [248]. Other studies to optimize and enhance vaccination efficacy suggested intradermal, high-dose, and oil-in-water emulsion approaches containing MF59 adjuvants. In older adults, these formulations have slightly more immunogenicity. A meta-analysis of studies in older adults revealed that IV administration of high-dose vaccines was significantly more effective in reducing major cardiopulmonary events compared to standard-dose vaccines [249].

Immunomodulatory nanosystems (IMNs) have shown excellent preclinical results in the treatment of various inflammatory diseases (IDs), but several challenges must be addressed before their clinical application. IMNs are engineered to actively modulate immune responses, for example, by incorporating cytokines, TLR ligands, or checkpoint inhibitors that direct dendritic cell polarization or T-cell activation. Furthermore, IMNs are designed to selectively suppress the body's innate immune system by depleting or inhibiting the physiological effects of HIC (hyperactive immune cells). It has a short-term therapeutic effect and causes a temporary immunodeficiency of the body, making it susceptible to various opportunistic infections. However, some IMNs failed to demonstrate the expected immunomodulatory effects in humans during clinical trials. There is a need to understand how the physicochemical properties of IMNs, such as size, charge, morphology, composition, may influence specific immune responses. Moreover, the targeting approaches that allow controlled delivery are highly desirable to advance the IMN concept in the treatment of IDs. The immunomodulatory approaches can also be combined with traditional drug delivery approaches to develop more advanced combinatorial platforms [250]. Nano-immunotherapy has also been shown to halt plaque progression, highlighting the potential of this approach for the acute treatment of atherosclerosis [251]. Mapping the cellular and molecular compositions of atherosclerotic tissues using single-cell technologies, such as CyTOF and single-cell RNA sequencing, holds the potential to broaden our understanding of the intricate immune mechanisms that directly cause atherosclerosis. These tools help build an immune atlas for revealing novel immune abnormalities [252]. Although sleep is important for immune function, the systematic study of the interaction between sleep and the immune system is a newly established field of research. As a result, many unknowns still need to be investigated in this field. It is likely that various parameters act in concert to mediate the effects of sleep on the immune system and vice versa [253]. Autoimmunity to LDL offers a unique route for developing innovative vaccination approaches [254,255]. The B and T cells have a more significant impact on the adaptive immune responses in atherosclerosis. It may be interesting to use T follicular regulatory (TFR) cells in the primary treatment of patients with familial hypercholesterolemia [256]. While the specific metabolomics of macrocyclic compounds like cucurbiturils (CB), calixarenes, pyrazines, and cyclodextrins (CyD) are not fully understood, there is optimism regarding their potential future role in immunotherapy [257]. Clinical imaging biomarkers for increased stroke risk may be based on systemic and neuroinflammatory changes, leading to personalized treatment of inflammation. The findings also encourage clinical research that utilizes whole-body PET/MRI to evaluate inflammation [258]. By harnessing the power of immunization to target specific antigens associated with plaque formation, researchers envision a future where vaccination could become a cornerstone of cardiovascular health maintenance.

## 7 Conclusion

The future of atherosclerosis vaccination hinges on strategically targeting antigens central to plaque pathogenesis, leveraging advances in immunology and delivery technologies. Preclinical evidence highlights PCSK9 and ApoB-100-derived peptides (e.g., P210), consistently reducing plaque burden and LDL-cholesterol in animal models via antibody-mediated inhibition of lipid uptake and inflammation. Concurrently, vaccines targeting inflammatory mediators (e.g., IL-1β, IL-12, and CX3CL1/CCL2) reduce monocyte recruitment and vascular inflammation, while extracellular matrix stabilizers (e.g., ADAMTS-7) enhance plaque integrity by modulating collagen metabolism and promoting immune tolerance. Nanoparticle delivery systems (e.g., liposomal, PLGA-based carriers) significantly enhance vaccine efficacy by improving antigen stability, immunogenicity, and targeted immune modulation. However, translation to humans requires rigorous optimization of antigen selection (prioritizing self-antigens with low autoimmunity risk), delivery routes (e.g., intranasal, nanocarrier-facilitated), and long-term safety profiles. Future clinical trials must validate these strategies, focusing on combinatorial approaches that address both lipid metabolism and inflammation to mitigate atherosclerosis in high-risk populations, particularly those with autoimmune comorbidities.

## Abbreviations

op/op  Osteopetrotic mice

LDL Low-density lipoprotein

CD8^+^ Cluster of Differentiation 8

Th1 Type 1 T helper

Th2 Type 2 T helper

Th17 Type 17 T helper

VSMC Vascular smooth muscle cells

LDLR −/− Low-density lipoprotein receptor-deficient mice

CD137 Tumor necrosis factor receptor superfamily member 9 (TNFRSF9) also known as CD137

CBL-B Casitas B-lineage lymphoma B inhibitor

IgG Immunoglobulin G

muMT- mice: Homozygous mutant mice lack mature B cells

DCs Dendritic cells

pDCs Plasmacytoid dendritic cells

cDCs Conventional dendritic cells

CCL17^+^ CC chemokine ligand 17 OR Thymus and activation-regulated chemokine (TARC)

Atg16l1 Autophagy related 16 Like 1

MMPs Matrix metalloproteinase

ox-LDL Oxidized LDL

VCAM-1 Vascular cell adhesion molecule-1

ICAM-1 Intercellular adhesion molecule-1

PDGF Platelet-derived growth factor

T1DM Type 1 diabetes mellitus

ADAMTS-7 A disintegrin and metalloproteinase with thrombospondin motifs 7

PCSK9 Proprotein convertase subtilisin/kexin type 9

TIE2:Ligands of the endothelial-enriched tunica interna endothelial cell kinase 2.

MDA-LDL Circulating malondialdehyde-modified LDL

HSP60 Eukaryotic heat shock protein 60

LDLR Low-density lipoprotein receptor

KLH Keyhole limpet hemocyanin

DDS Drug delivery strategies

PLGA NPs Poly (lactic-*co*-glycolic acid) nanoparticles

MF59 An immunologic adjuvant that uses squalene

IMNS Integrated Micro and Nano Systems

IDs Inflammatory diseases

IgM: Immunoglobulin M

HCT Hematocrit

CyTOF Cytometry by the time of flight

AS Ankylosing spondylitis

PET/CT A procedure that combines the pictures from a positron emission tomography (PET) scan and a computed tomography (CT) scan

Doppler A noninvasive test that can be used to measure the blood flow through your blood vessels; HDF: High-fat diet

TFH cells T follicular helper cells

MZB cells Marginal-zone B cells

CB Cucurbituril (269) is a nonadecacyclic cage compound

CyD Cyclodextrins

PET/MRI: Positron emission tomography–magnetic resonance imaging

HIC Hyperactive immune cell

SOCS3 A tumor suppressor of cytokine signaling

SMCs Smooth muscle cells

LDL-C Low-density lipoprotein cholesterol

NOSs Nitric oxide synthases

PBS Phosphate-buffered saline

COMP Cartilage oligomeric matrix protein

LRP1 Lipoprotein receptor-related protein 1

Wnt5a Wnt Family Member 5 A

ABCA1 ATP binding cassette transporter A1

NCEH1 Neutral cholesterol ester hydrolase

IRAK-1 Interleukin-1 receptor-associated kinase-1

NZW Female New Zealand White rabbits

CX3CL1 Fractalkine- chemokine (C-X3-C motif) ligand 1

CX3CR1 Fractalkine receptor- CX3C motif chemokine receptor 1

scFv Single chain fragment variable

DEC205-CD205- MMR Macrophage mannose receptor

CCL2 Chemokine (C—C motif) ligand 2

GPNMB Glycoprotein nonmetastatic melanoma protein B

SRs Scavenger receptors

MSR Macrophage scavenger receptor

SRA Scavenger Receptor A

CCBs Calcium channel blockers

ARBs Angiotensin receptor blockers

ACE Angiotensin-converting enzyme

GPCRs-7TM G protein-coupled receptors-7-Transmembrane receptors

ERK Extracellular signal-regulated kinase

AT1R Angiotensin II type 1 receptor

RAS Reticular activating system

AngII Angiotensin II

ROS Reactive oxygen species

DNGR1 Dendritic cell NK lectin group receptor-1

TFR cells T follicular regulatory cells

Qβ-VLP Qβ bacteriophage virus-like particle

HDL-C HDL-cholesterol

BH4, THB tetrahydrobiopterin

Tregs Regulatory T cells

## CRediT authorship contribution statement

**Alireza Haraj:** Investigation, Data curation. **Masoomeh Bakhshandeh:** Writing – original draft, Investigation, Data curation. **Nafiseh Shokri:** Visualization. **Prand Shariat:** Investigation, Data curation. **Ali Alyan:** Investigation, Data curation. **Zahra Chegini:** Investigation, Data curation. **Mohammad Ali Nazari:** Investigation, Data curation, Conceptualization. **Seyed Parsa Seyedi Taji:** Investigation, Data curation. **Mohammad Najafi:** Writing – original draft, Supervision, Conceptualization.

## Consent for publication

No applicable.

## Ethics approval and consent to participate

No applicable.

## Funding

No applicable.

## Declaration of competing interest

No applicable.

## Data Availability

No applicable.
